# Comment on ‘Effects of Zinc Supplementation on Glycemic Control, Insulin Resistance, Inflammation and Oxidative Stress in Diabetes’

**DOI:** 10.1002/edm2.70328

**Published:** 2026-08-31

**Authors:** Héctor Fuentes‐Barría

**Affiliations:** ^1^ Centro de Investigación en Medicina de Altura (CEIMA) Universidad Arturo Prat Iquique Chile

To the Editor,

I read with interest the article by Loaiza‐Giraldo et al. [1], recently published, evaluating the effects of zinc supplementation on metabolic, inflammatory and oxidative stress outcomes in individuals with diabetes. I consider this to be a clinically relevant topic; however, several methodological aspects may limit the interpretation.

First, the authors state in their review that eligible participants were individuals with type 1 or type 2 diabetes mellitus; however, studies involving individuals with gestational diabetes and prediabetes were subsequently included. Similarly, the corresponding PROSPERO record defines the eligible population as ‘subjects with diabetes mellitus’, without explicitly specifying these additional populations [1]. These conditions differ substantially in terms of pathophysiology, baseline risk and clinical management [2]. Therefore, their combined inclusion may increase clinical heterogeneity and compromise the applicability of the pooled estimates.

Second, some interventions did not consist exclusively of zinc supplementation but included other micronutrients or bioactive compounds. The authors themselves acknowledge that these co‐interventions make it difficult to attribute the observed effects specifically to zinc [1]. In this context, the combined inclusion of zinc‐only interventions and multicomponent interventions could introduce clinical heterogeneity and complicate the interpretation of the pooled effects. Therefore, sensitivity analyses excluding multicomponent interventions would be advisable to assess the extent to which the observed results can be specifically attributed to zinc supplementation and to determine the robustness of the pooled estimates [3].

Third, several pooled outcomes showed substantial or considerable statistical heterogeneity. For example, plasma zinc concentrations showed an *I* ^2^ of 99%, while other metabolic and inflammatory outcomes also demonstrated high heterogeneity. Although random‐effects models and meta‐regression were applied, substantial residual heterogeneity remained [1]. Under these circumstances, the clinical meaning and generalizability of a single pooled estimate should be interpreted cautiously.

Fourth, the authors performed multiple meta‐regression analyses involving age, sex, zinc dose and intervention duration, despite several outcomes being based on a limited number of studies [1]. This is particularly concerning because meta‐regression generally requires an adequate number of studies per moderator, and the Cochrane Handbook advises caution when fewer than 10 studies are available. Moreover, methodological research has shown that meta‐regression analyses are frequently affected by overfitting and other methodological pitfalls that may lead to misleading findings. Therefore, given the limited number of studies and the multiple moderators examined, these associations should be interpreted as exploratory and hypothesis‐generating rather than confirmatory [4].

Finally, the clinical interpretation of the findings warrants caution. The meta‐analysis did not demonstrate significant improvements in fasting plasma glucose or HbA1c, despite the title emphasizing ‘glycemic control’. In contrast, significant effects were observed primarily for surrogate markers such as insulin, HOMA‐IR, CRP and oxidative stress biomarkers [1]. These statistically significant changes should therefore not necessarily be equated with clinically meaningful improvements in diabetes control or patient‐important outcomes [5].

Taken together, these issues warrant caution when attributing the pooled effects specifically to zinc supplementation. Stratified and sensitivity analyses by diabetes phenotype, zinc‐only interventions, and clinically relevant glycemic outcomes could strengthen the validity and clinical interpretation of the conclusions.

## Author Contributions

**Héctor Fuentes‐Barría:** conceptualization, investigation, writing – original draft, methodology, writing – review and editing, formal analysis, resources.

## Funding

The author has nothing to report.

## Ethics Statement

The author has nothing to report.

## Consent

The author has nothing to report.

## Conflicts of Interest

The author declares no conflicts of interest.

## Data Availability

The author has nothing to report.
